# Soybean continuous cropping affects yield by changing soil chemical properties and microbial community richness

**DOI:** 10.3389/fmicb.2022.1083736

**Published:** 2022-12-30

**Authors:** Yan Li, Chuanqi Shi, Dan Wei, Xuejia Gu, Yufeng Wang, Lei Sun, Shanshan Cai, Yu Hu, Liang Jin, Wei Wang

**Affiliations:** ^1^Resources and Environment, Beijing Academy of Agriculture and Forestry Sciences, Institute of Plant Nutrition, Beijing, China; ^2^Heilongjiang Province Key Laboratory of Cold Region Wetland Ecology and Environment Research, Harbin University, Harbin, Heilongjiang, China; ^3^Heilongjiang Academy of Black Soil Conservation and Utilization, Heilongjiang Academy of Agricultural Sciences, Harbin, Heilongjiang, China; ^4^College of Resource and Environment, Northeast Agricultural University, Harbin, Heilongjiang, China

**Keywords:** black soil, soybean continuous cropping, bacteria, fungi, yield

## Abstract

In agroecosystems, different cropping patterns cause changes in soil physicochemical properties and thus in microbial communities, which in turn affect crop yields. In this study, the yields of soybean continuous cropping for 5 years (C5), 10 years (C10), and 20 years (C20) and of soybean-corn rotational cropping (R) treatments were determined, and samples of the tillage layer soil were collected. High-throughput sequencing technology was used to analyze the diversity and composition of the soil bacterial and fungal communities. The factors influencing microbial communities, along with the effects of these communities and those of soil chemical indexes on yield, were further evaluated. The results showed that the community richness index of bacteria was higher in C20 than in R and that of fungi was highest in C5. The differences in the bacterial and fungal communities diversity indexes were not significant among the different continuous cropping treatments, respectively. The soil microbial community composition of all continuous cropping treatments differed significantly from R. The dominant bacterial phylum was Actinobacteriota and the dominant fungal phylum was Ascomycota. The relative abundance of *Fusarium* did not differ significantly among the continuous cropping treatments, while that of the plant pathogen fungi *Lectera* sp., *Plectosphaerella* sp., and *Volutella* sp. increased with continuous cropping years. Soil pH, SOM, N, and TP had significant effects on both bacterial and fungal communities, and TK and C/N had highly significant effects on fungal communities. The yield of C5 was significantly lower than that of R, and the differences in yield between C10, C20, and R were not significant. TN, TP, and pH had significant effects on yield, and fungal community abundance had a greater negative effect on yield than bacterial community abundance.

## Introduction

The black soil (Mollisol) region of northeastern China is a major soybean [*Glycine max* (L.) Merrill] production area, where soybean continuous cropping is common. Soybean is considered a typical crop sensitive to continuous cropping, which leads to lower yields and poorer quality (Liu et al., 2012; Wang et al., 2012). The reason for this is the increase in the number of pathogenic fungi and the decrease in the number of beneficial fungi, as well as the decrease in bacterial density, i.e., the change in the soil microbial community structure from “bacterial type” to “fungal type” (Ma et al., 1997). For example, the increased abundance of the pathogenic fungi *Fusarium*, *Thelebolus* and *Mortierellales* can cause root rot in soybean to increase (Bai et al., 2015). Song et al. (2018) proposed that soil fertility and microbial community stability were improved by changing continuous cropping patterns and using soybean in rotation with corn or wheat and that diversified cropping patterns could increase soil productivity and soybean yield. Later, Song et al. (2022) reported that, compared to fallow plots, soybean continuous cropping caused the relative abundance of Proteobacteria, Bacteroidetes, Chloroflexi, etc., to decrease and that of Actinobacteriota, Gemmatimonadetes, Planctomycetes, etc., to increase. Additionally, *Fusarium* abundance after 13 years of soybean continuous cropping was significantly higher than that in fallow plots and in corn continuous cropping and increased the incidence of root rot. Liu H. et al. (2019)) and Liu H. et al. (2020) suggested that the reason for the obstacles encountered in soybean continuous cropping is the higher relative abundance and community diversity of potential plant pathogenic fungi (e.g., *Fusarium*, *Cylindrocarpon*, *Gibberella*) in long-term (27 years) continuous cropping soils than in rotational cropping soils, creating a fungal community structure unfavorable to soybean growth.

However, Wei et al. (2015) showed that in the black soil region of northeastern China, long-term (20 years) continuous cropping was able to reduce soybean root diseases, and the population density of pathogenic fungi (*Fusarium* spp. and *Heterodera glycines*) was significantly lower than that in soil under soybean in rotation with wheat or with corn. Moreover, higher levels of biological control agents were found under long-term continuous cropping, which can form a soil microbial community that inhibits the occurrence of soybean root diseases. Chen et al. (2018) showed that at 7 years of soybean continuous cropping, the soil bacterial community composition and diversity were significantly lower than those in soil under rotational cropping or under less than 4 years of continuous cropping. Additionally, the fungal community in the long-term continuous cropping treatment had high similarity to that in the rotational cropping treatment, and there was a tendency for disease to diminish in the former, indicating the formation of disease suppressive soils. Liu J. et al. (2019) showed that soybean long-term (17 years) continuous cropping led to an increasing abundance of pathogenic fungi in the soil, along with an increasing abundance of some beneficial fungi, which suggested that suppressive soils might be developed after long-term continuous cropping. Liu Z. et al. (2019) and Liu Z. et al. (2020) similarly concluded that the relative abundance of the pathogenic fungi *Fusarium* sp. was reduced by soybean long-term (13 years) continuous cropping or soybean-corn rotational cropping (5 years), which could alleviate the obstacle posed by soybean continuous cropping.

Soil microbes, as decomposers in ecosystems, can degrade plant litter, participate in soil nutrient cycling, contribute to soil development, play an important role in maintaining soil ecosystem stability (Toor and Adnan, 2020; Prasad et al., 2021) and can be used as indicators of soil ecosystem health and productivity (Dias et al., 2015; Pervaiz et al., 2020). Soil microbial community composition and diversity are directly or indirectly influenced by chemical indexes such as pH, electrical conductivity, and nutrient content (Wang et al., 2019; Shi et al., 2021). In agroecosystems, soybean continuous cropping increases soil pH, which significantly affects soil bacterial communities, while the ratio of carbon (C) and nitrogen (N) has a significant effect on fungal communities (Liu J. et al., 2019; Liu Z. et al. (2020)). Changes in both soil physicochemical properties and beneficial or harmful microbial abundance can affect crop yields directly, and changes in soil microbial communities caused by changes in physicochemical properties can affect yields indirectly (Liu et al., 2016; Strom et al., 2020).

Some studies have reported on soil microbial communities under different soybean cropping patterns in black soil regions, but the factors influencing them and the relationship between them and soybean yield are still not clear. Therefore, in this study, we collected soil from the tillage layer (0–20 cm) of soybean under different continuous cropping years (5, 10, and 20 years) and of soybean-corn rotational cropping (25 years) in the black soil region of northeastern China with the objectives of (1) revealing the diversity and compositional characteristics of soil bacteria and fungi, (2) determining the key influencing factors of soil bacterial and fungal communities, and (3) evaluating the effects of the key influencing factors on yield.

## Materials and methods

### Experimental area description

The experiment was conducted in the Modern Agricultural Innovation Park (126°50′ E, 45°49′ N) in Minzhu Township, Harbin City, China. The experimental area was flat and the soil was typical black soil. Long-term continuous cropping of soybean and soybean-corn rotational cropping in the experimental field with an annual monoculture system began in 1995. The variety of soybean was Heinong 48. The annual fertilizer application rates were 45 kg·ha^−1^ N, 90 kg·ha^−1^ P_2_O_5_, and 45 kg·ha^−1^ K_2_O, and all fertilizers were applied at once as a base fertilizer.

### Soybean yield determination

Yield determination was conducted in September 2019, during soybean harvest, in 10 small 5 m^2^ plots within the sample plots of 5 (C5), 10 (C10), and 20 (C20) years of continuous cropping and the sample plot of soybean-corn rotational cropping (R). The yield determination was replicated three times.

### Soil sampling and chemical index determination

Three sample plots (2 × 2 m) were set up for each treatment as three replications. Five points (apex and center) were used for sampling, and 200 g of bulk soil was collected from each point in the tillage layer (0–20 cm). In the laboratory, soil pH was determined (water:soil = 2.5:1) using a composite electrode method (INESA PHS–3C, Shanghai, China); soil organic matter (SOM) content was determined by the potassium dichromate oxidation-ferrous sulfate titration method; dissolved organic C (DOC) content was determined using a TOC analyzer (Analytik Jena Multi N/C 2100, Germany); total N (TN) content was determined by the semimicro Kjeldahl method; total P (TP) content was determined by the molybdenum antimony colorimetric method; total K (TK) content was determined by the flame spectrophotometry method; alkali-hydrolyzed N (AN) content was determined by the alkaline diffusion method; available P (AP) content was determined by the sodium bicarbonate extraction-molybdenum antimony anticolorimetric method; available K (AK) content was determined by the ammonium acetate extraction-flame photometric method; and ammonium N (NH_4_^+^–N) content and nitrate N (NO_3_^−^–N) content were determined by a flow analyzer (Bran Luebbe, Germany) after extraction with 1 mol·L^−1^ KCl (Lu, 2000; Shi et al., 2021). All index measurements were repeated three times.

### Soil sample DNA extraction

Taking 0.5 g of soil sample, the total DNA was extracted using an E.Z.N.A.® soil DNA Kit (Omega Bio-Tek, Norcross, GA, United States) following the manufacturer’s instructions. A Nano Drop 2000 UV–Vis spectrophotometer (Thermo Fisher Scientific, Wilmington, DE, United States) was used to detect the DNA concentration and purity. Then 1% agarose gel electrophoresis was used for DNA quality testing.

### PCR amplification and sequencing

PCR amplification was performed using bacterial primer 338F_806R (338F: 5′–ACTCCTACGGGAGGCAGCAG–3′ 806R: 5′–GGACTACHVGGGTWTCTAAT–3′) and fungal primer ITS1F_ITS2R (ITS1F: 5′–CTTGGTCATTTAGAGGAAGTAA–3′ ITS2R: 5′–GCTGCGTTCTTCATCGATGC–3′). Following Liu Z. et al. (2020), the volume of the PCR system was 20 μL, containing 4 μL 5 × Fast*Pfu* Buffer; 2 μL 2.5 mM dNTPs; 0.8 μL of each primer for a final concentration of 5 μM; 0.4 μL Fast*Pfu* Polymerase; 0.2 μL BSA; 10 ng template DNA; and ddH_2_O to complete 20 μL. The PCR parameters were as follows: (1) 3 min, 95°C denaturation. (2) 30 s, 95°C; 30 s, 55°C annealing; 45 s, 72°C elongation, 35 cycles. (3) 10 min, 72°C extension. The PCR products were detected by 2% agarose gel electrophoresis and recovered using a DNA gel recovery kit (Axygen Biosciences, Union City, CA, United States). The PCR products were quantified by the Quanti Fluor™–ST Blue Fluorescence Quantification System (Promega, Madison, WI, United States). Samples were sent to Majorbio Biopharmaceutical Technology Co., Ltd. (Shanghai, China) for sequencing using the Illumina MiSeq high-throughput sequencing platform (Illumina, San Diego, CA, United States). Each sample was analyzed in triplicate.

### Data processing

MiSeq sequencing obtained paired-end reads, and optimized data were obtained by using Fastp v0.19.6 and FLASH v1.2.11 software (Yang et al., 2019). All data were submitted to the NCBI Sequence Read Archive database (Accession number: bacteria PRJNA887719 and fungi PRJNA887732). Operational taxonomic unit (OTU) clustering of non-repeated sequences was performed at 97% similarity using the UPARSE pipeline^1^ (Edgar, 2013). The taxonomic analysis of OTUs was performed by applying the Ribosomal Database Project Classifier and the Bayes algorithm with a 0.7 confidence level (Wang et al., 2007), and the taxonomic identification databases were SILVA 138/16s bacteria (Quast et al., 2013) and UNITE 8.0/its fungi 2. The relative abundance of each OTU was counted.

The Chao1 richness index and Shannon diversity index of the microbial community of each sample were analyzed using Mothur v1.39.5 software. Analysis of variance (ANOVA) was performed using SPSS 17.0 (SPSS Inc. Chicago, IL, United States) to analyze the significance of differences in yield, soil chemical indexes, community richness and diversity indexes, and relative abundance of taxa among treatments. Bar plots were drawn using Office Excel 2016 based on the relative abundance of dominant taxa. On the Majorbio Cloud Platform (Ren et al., 2022), non-metric multidimensional scaling (NMDS) analysis was performed based on Bray-cutis distance at the OTU level, and the significance of differences was tested using Adonis (999 permutations). Linear discriminant analysis effect size (LEfSe) was performed using the non-parametric factorial Kruskal-Wallis sum-rank test to detect taxa with significant differences in relative abundance among treatments, and linear discriminant analysis (LDA) was used to estimate the size of the effect of each taxon on the difference in relative abundance. The Mantel test was used to identify chemical indexes with significant effects on soil bacterial and fungal communities. Redundancy analysis (RDA) and variance partitioning analysis (VPA) were used to evaluate the degree of influence. Fungal trophic modes were classified and their functional taxa were predicted using FUNGuild v1.0 (Nguyen et al., 2015). A structural equation model was fitted using IBM SPSS Amos 26 graphics to evaluate the effects of bacterial and fungal communities and chemical indexes on yield.

## Results

### Soybean yield

Based on the yield determination, using one-way ANOVA and Duncan’s multiple comparison test, the differences in yields among C10, C20 and R were not significant (*p* > 0.05). However, the yields of C10, C20, and R were significantly (*p* < 0.05) higher than that of C5 by 9.11, 7.30 and 6.08%, respectively (Table 1). The results indicated that the yields of long-term continuous cropping were significantly higher than those of short-term continuous cropping and were close to those of R.

**Table 1:** Soybean yield.

Treatments	C5	C10	C20	R
Yield (kg·ha^−1^)	2712.22 ± 67.98 b	2983.95 ± 45.10 a	2925.86 ± 62.28 a	2887.78 ± 42.05 a

Data represent the mean ± standard deviations, Analysis of variance (Duncan’s multiple comparison test) was used to test the significance of differences. The different lowercase letters represent significant differences (*p* < 0.05). C5, C10, and C20 represent soybean continuous cropping treatments for 5, 10, and 20 years, respectively, and R represents soybean-corn rotational cropping treatment.

### Soil chemical properties

As shown in Table 2, soil pH showed a significant decreasing trend with increasing years of continuous cropping and was significantly higher in R. The SOM content showed an increasing trend with increasing years of continuous cropping and was significantly lower in R. The DOC content was significantly lower in C10 than in C20 and R and was not significantly different from C5. The TN content was not significantly different between C5 and R, it increased significantly with increasing years of continuous cropping, and AN, NH_4_^+^–N and NO_3_^−^–N contents showed similar trends. The TP and TK contents showed a decreasing trend with increasing years of continuous cropping and were significantly lower in the former treatments than in R. The AP contents were not significantly different among the treatments, while the AK contents were significantly higher in C5 and C10 than in C20 and R. The C/N was not significantly different among the continuous cropping treatments but was significantly lower in the former treatments than in R.

**Table 2:** Soil chemical indexes of soybean continuous and rotational cropping.

Indexes	C5	C10	C20	R
pH	5.80 ± 0.01 b	5.66 ± 0.01 c	5.61 ± 0.02 d	6.01 ± 0.02 a
SOM (g·kg^−1^)	22.19 ± 0.96 c	26.68 ± 0.72 b	27.79 ± 0.92 b	31.08 ± 0.57 a
DOC (g·kg^−1^)	0.24 ± 0.01 ab	0.23 ± 0.01 b	0.26 ± 0.02 a	0.25 ± 0.01 a
TN (g·kg^−1^)	0.83 ± 0.03 c	1.00 ± 0.01 b	1.11 ± 0.04 a	0.82 ± 0.01 c
AN (mg·kg^−1^)	190.81 ± 4.53 b	205.06 ± 4.15 a	208.93 ± 3.01 a	187.52 ± 2.80 b
NH_4_^+^–N (mg·kg^−1^)	20.10 ± 0.40 d	37.87 ± 0.47 b	45.81 ± 1.02 a	25.00 ± 1.30 c
NO_3_^−^–N (mg·kg^−1^)	7.84 ± 0.82 d	17.94 ± 0.82 b	21.09 ± 0.75 a	10.32 ± 0.21 c
TP (g·kg^−1^)	1.26 ± 0.01 b	1.17 ± 0.02 c	1.02 ± 0.03 d	1.38 ± 0.01 a
AP (mg·kg^−1^)	52.28 ± 2.32 a	49.99 ± 3.92 a	51.07 ± 0.95 a	52.76 ± 2.69 a
TK (g·kg^−1^)	25.91 ± 0.31 b	25.89 ± 1.07 b	22.90 ± 0.55 c	32.60 ± 1.19 a
AK (mg·kg^−1^)	189.49 ± 1.56 a	187.48 ± 0.51 a	178.74 ± 8.54 b	176.21 ± 0.96 b
C/N	15.44 ± 0.38 b	15.36 ± 0.35 b	14.45 ± 0.87 b	21.80 ± 0.31 a

Data represent the mean ± standard deviations, Analysis of variance (Duncan’s multiple comparison test) was used to test the significance of differences. The different lowercase letters represent significant differences (*p* < 0.05). C5, C10, and C20 represent soybean continuous cropping treatments for 5, 10, and 20 years, respectively, and R represents soybean-corn rotational cropping treatment. SOM, soil organic matter; DOC, dissolved organic C; TN, total N; AN, alkali-hydrolyzed N; NH_4_^+^–N, ammonium N; NO_3_^−^–N, nitrate N; TP, total P; AP, available P; TK, total K; AK, available K; C/N, ratio of C and N.

### Microbial community diversity

The microbial raw sequences were quality-controlled and filtered to obtain 542,095 and 848,233 valid sequences for cluster analysis of bacterial and fungal OTUs, respectively. The total number of OTUs obtained by clustering was 4,407 and 1,123, and the core OTUs (the common OTUs to all treatments) accounted for 49.33% (2174) and 19.86% (223) of the total for bacteria and fungi, respectively. The number of soil bacterial species was significantly lower in C5 and C10 than in C20, while the difference between continuous cropping and R was not significant. The number of fungal species was significantly higher in C5 than in C10, C20, and R (Table 3).

**Table 3:** Diversity indexes of soil bacterial and fungal communities.

Treatments	Bacteria			Fungi		
	Sobs	Chao1	Shannon	Sobs	Chao1	Shannon
C5	2,365 ± 14 b	3217.67 ± 35.32 ab	6.09 ± 0.11 b	456 ± 47 a	486.38 ± 53.84 a	3.90 ± 0.28 a
C10	2,373 ± 70 b	3183.33 ± 71.49 b	6.12 ± 0.02 ab	336 ± 29 b	349.63 ± 32.46 b	3.86 ± 0.19 a
C20	2,540 ± 139 a	3354.37 ± 124.74 a	6.19 ± 0.04 ab	360 ± 23 b	376.60 ± 22.38 b	4.03 ± 0.03 a
R	2,419 ± 53 ab	3111.85 ± 43.66 b	6.24 ± 0.01 a	339 ± 28 b	351.65 ± 23.26 b	4.23 ± 0.13 a

Data represent the mean ± standard deviations. Analysis of variance (Duncan’s multiple comparison test) was used to test the significance of differences. The different lowercase letters represent significant differences (*P* < 0.05). C5, C10, and C20 represent soybean continuous cropping treatments for 5 years, 10 years, and 20 years, respectively, and R represents soybean-corn rotational cropping treatment.

In the soil bacterial community, the Chao1 index was significantly higher in C20 than in C10 and R, and the difference between C5 and each treatment was not significant. The Shannon index tended to increase with increasing years of continuous cropping, but the difference was not significant, with C5 being significantly lower than R. In the soil fungal community, the Chao1 index was significantly higher in C5 than in C10, C20, and R; the Shannon index of R was high, but the differences were not significant among treatments.

### Microbial community composition

**Figure 1:**
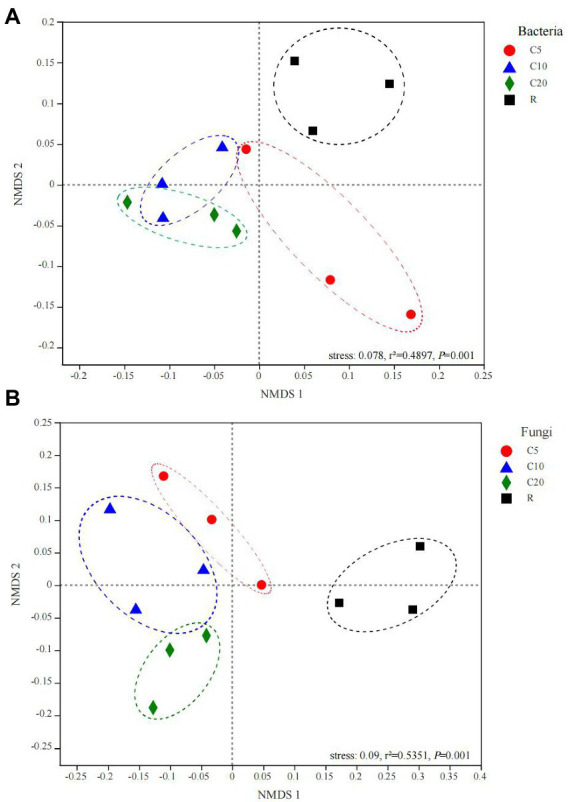
NMDS analysis among soil bacterial **(A)** and fungal **(B)** samples of different treatments based on OTU level. The significance of differences was tested using Adonis (999 permutations). C5, C10, and C20 represent soybean continuous cropping treatments for 5, 10, and 20 years, respectively, and R represents soybean-corn rotational cropping treatment.

Based on NMDS analysis at the OTU level for the soil bacterial (Figure 1A) and fungal (Figure 1B) community compositions, R was clearly separated from continuous cropping. The bacterial communities of C10 and C20 were closer than those of C5 on the NMDS 1 and NMDS 2 axes; the fungal communities of C5, C10 and C20 were closer on NMDS 1, and those of C5 and C10 were closer on NMDS 2.

**Figure 2:**
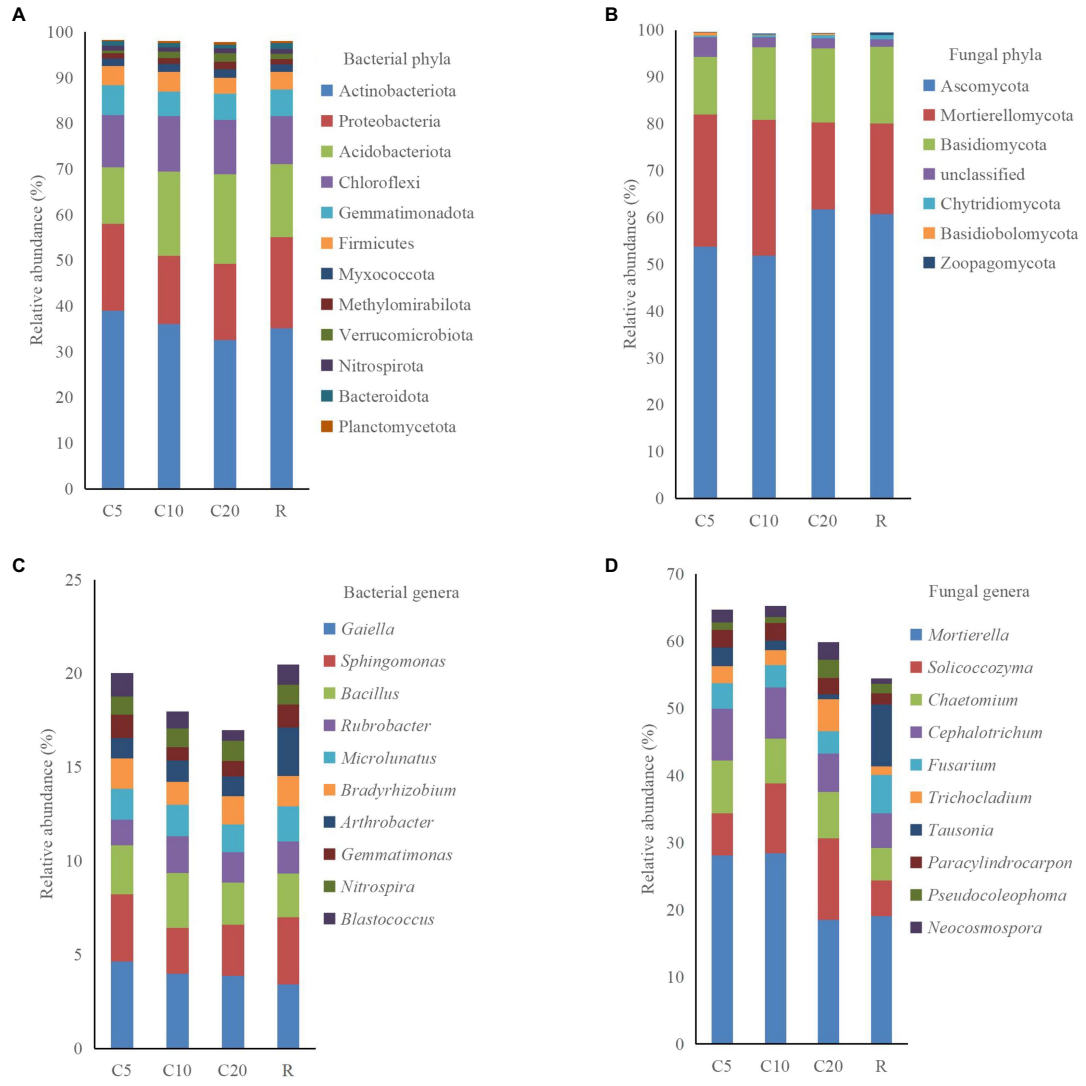
Relative abundance of dominant phyla and genera (top 10 except unclassified taxa) of soil bacteria and fungi. C5, C10, and C20 represent soybean continuous cropping treatments for 5, 10, and 20 years, respectively, and R represents soybean-corn rotational cropping treatment. **(A)** Bacterial phyla, **(B)** Fungal phyla, **C** Bacterial genera, and **D** Fungal genera.

A total of 37 phyla, 120 classes, 267 orders, 394 families, 677 genera, and 1,423 species were obtained for the bacterial community, and 14 phyla, 40 classes, 88 orders, 196 families, 356 genera, and 503 species were obtained for the fungal community. Twelve and seven phyla had relative abundances >0.5% in the bacterial and fungal communities, respectively. Based on Supplementary Table S1, the sum of the relative abundances of the first six phyla was >90% in the bacterial community (Figure 2A). The relative abundance of Actinobacteriota, Proteobacteria, and Gemmatimonadota differed among the treatments, but that of Acidobacteriota, Chloroflexi, and Firmicutes did not. The sum of the relative abundances of Ascomycota, Mortierellomycota, and Basidiomycota was close to 95% in the fungal community (Figure 2B). The difference in the relative abundance of Ascomycota between C20 and R was not significant, but both were significantly higher than C5 and C10. The relative abundances of Chytridiomycota, Basidiobolomycota, and Zoopagomycota were all below 1% in the treatments, while no Basidiobolomycota was found in R.

At a relative abundance level > 0.5%, the bacterial and fungal communities included 55 and 76 genera, respectively. The bacterial community had a high relative abundance of unclassified taxa in all treatments. The relative abundance of *Gaiella* was 4.65% in C5, which was significantly higher than that in R. The relative abundances of *Bacillus*, *Microlunatus*, and *Bradyrhizobium* were not significantly different among treatments (Figure 2C). In the fungal community, the relative abundances of *Mortierella*, *Chaetomium*, *Cephalotrichum*, *Paracylindrocarpon*, and *Pseudocoleophoma* did not differ significantly among treatments, i.e., the differences in the relative abundance of the dominant fungal genera were similar to the pattern of the dominant phyla among treatments. The relative abundance of *Fusarium* did not differ significantly among the continuous cropping treatments and was significantly lower in C10 and C20 than in R (Figure 2D).

### Significantly different taxa between treatments

**Figure 3:**
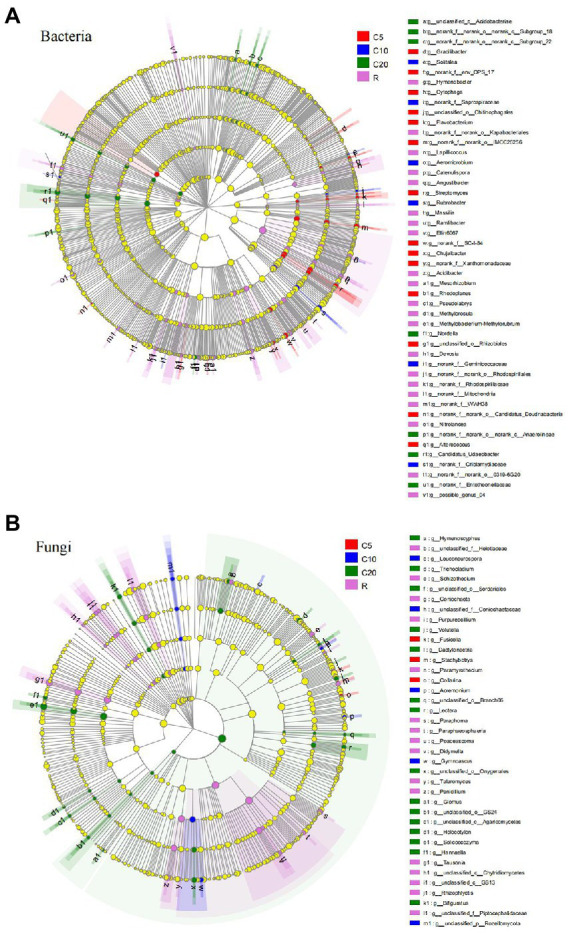
LEfSe analysis (LDA > 2) of soil bacterial **(A)** and fungal **(B)** communities. The levels of phylum, class, order, family and genus are arranged from the inside to the outside, and the genera are shown in the figure. The yellow circle represents the taxa with no significant difference among the treatments. C5, C10, and C20 represent soybean continuous cropping treatments for 5, 10, and 20 years, respectively, and R represents soybean-corn rotational cropping treatment.

The cladogram showed that when the LDA value was 2.0, there were 115 bacterial taxa and 85 fungal taxa that were significantly different among the treatments, including 3 phyla, 9 classes, 20 orders, 35 families and 48 genera (accounting for 7.09% of the total number of bacterial genera) and 5 phyla, 8 classes, 14 orders, 19 families, and 39 genera (accounting for 10.96% of the total number of fungal genera). Among the 48 bacterial genera, 14 genera in C5, 6 genera in C10, 7 genera in C20, and 21 genera in R had significantly higher relative abundance (Figure 3A), for example, *Streptomyces* (LDA = 3.207), *Rubrobacter* (LDA = 3.523), *Nordella* (LDA = 2.828), and *Lapillicoccus* (LDA = 3.341). Among the 39 fungal genera, 3 genera in C5, 5 genera in C10, 15 genera in C20, and 16 genera in R had significantly higher relative abundance (Figure 3B), for example, *Fusicolla* (LDA = 3.558), *Gymnoascus* (LDA = 3.350), *Solicoccozyma* (LDA = 4.204), and *Tausonia* (LDA = 4.613).

### Fungal trophic mode classification and functional prediction

To avoid overinterpretation of the trophic mode classification and functional prediction of fungi by FUNGuild, only the taxa with confidence levels of probable and above and relative abundance >0.5% were retained. A total of 98 OTUs were obtained, of which 84 (85.71%) were affiliated with Ascomycota and seven (7.14%) were affiliated with Basidiomycota. As shown in Supplementary Figure S1, the trophic mode included saprotroph (53 OTUs), pathotroph (19 OTUs), symbiotroph (4 OTUs), saprotroph-pathotroph (10 OTUs), pathotroph-symbiotroph (2 OTUs), saprotroph-symbiotroph (6 OTUs) and saprotroph-pathotroph-symbiotroph (4 OTUs). The number of OTUs contained in different trophic modes did not differ significantly among the treatments, respectively.

**Figure 4:**
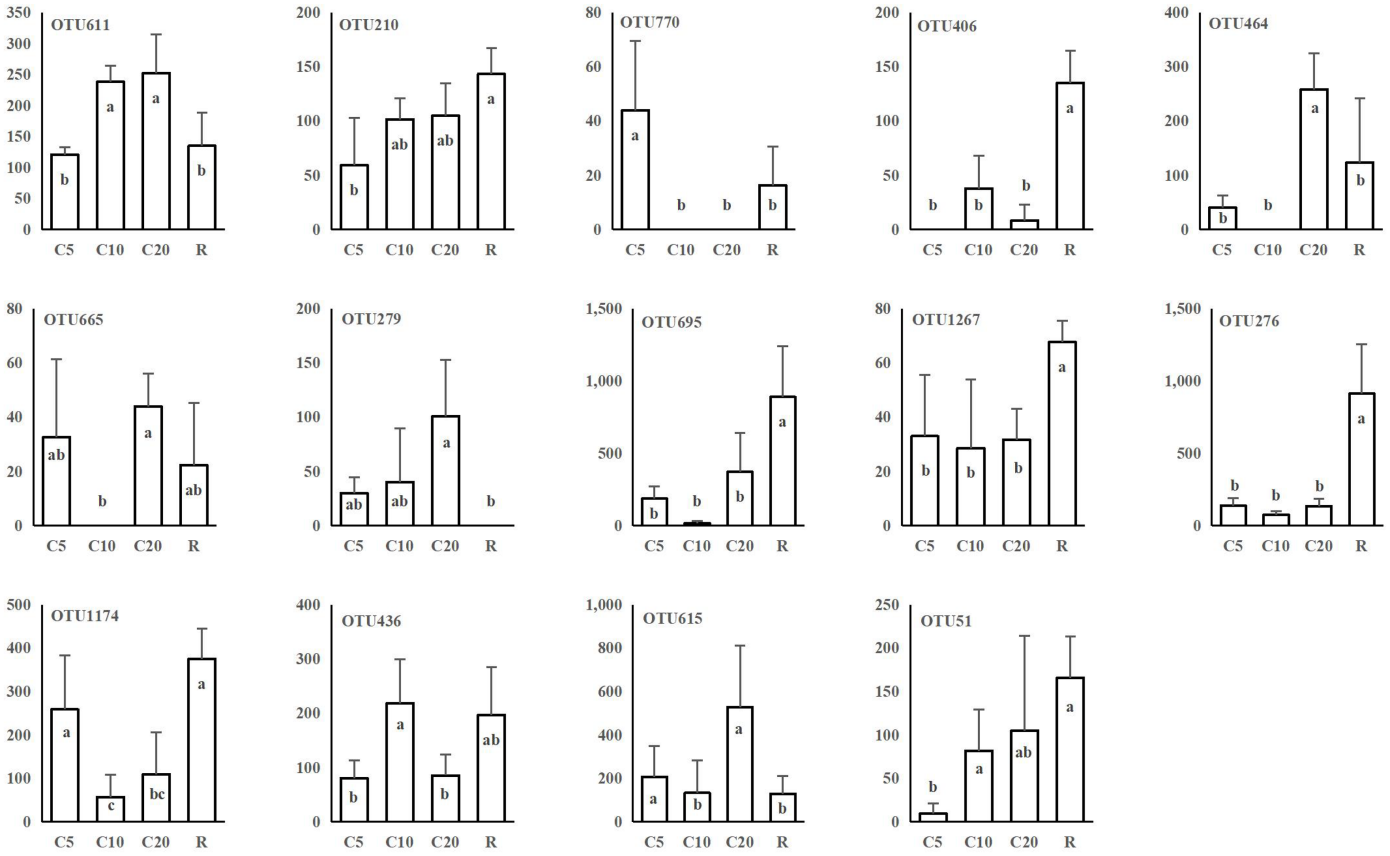
Plant-related OTUs with significant differences among the different cropping treatments. The vertical coordinate is the number of sequences in OTUs. Analysis of variance (Duncan’s multiple comparison test) was used to test the significance of differences. The different lowercase letters represent significant differences (*p* < 0.05). C5, C10, and C20 represent soybean continuous cropping treatments for 5, 10, and 20 years, respectively, and R represents soybean-corn rotational cropping treatment.

Plant-related fungi, such as leaf saprotrophic fungi, plant pathogenic fungi, and arbuscular mycorrhizal fungi, included a total of 40 OTUs belonging to 34 genera, of which 33 OTUs were affiliated with Ascomycota and four with Basidiomycota. Twenty-four OTUs were associated with plant pathogens, including *Neonectria candida*, *Gibberella intricans*, and *Gibellulopsis nigrescens*. The numbers of sequences of 14 OTUs associated with plants were significantly different among treatments (Figure 4), including 9 plant pathogenic OTUs. These results, combined with the information in Supplementary Table S2, indicated that continuous and rotational cropping changed the composition of functional fungi. OTU464, OTU665 and OTU279 contained a high number of sequences in C20, i.e., continuous cropping caused these OTUs to accumulate, while OTU695, OTU1267, and OTU276 were more abundant in R.

### Effect of soil chemical indexes on microbial communities

**Figure 5:**
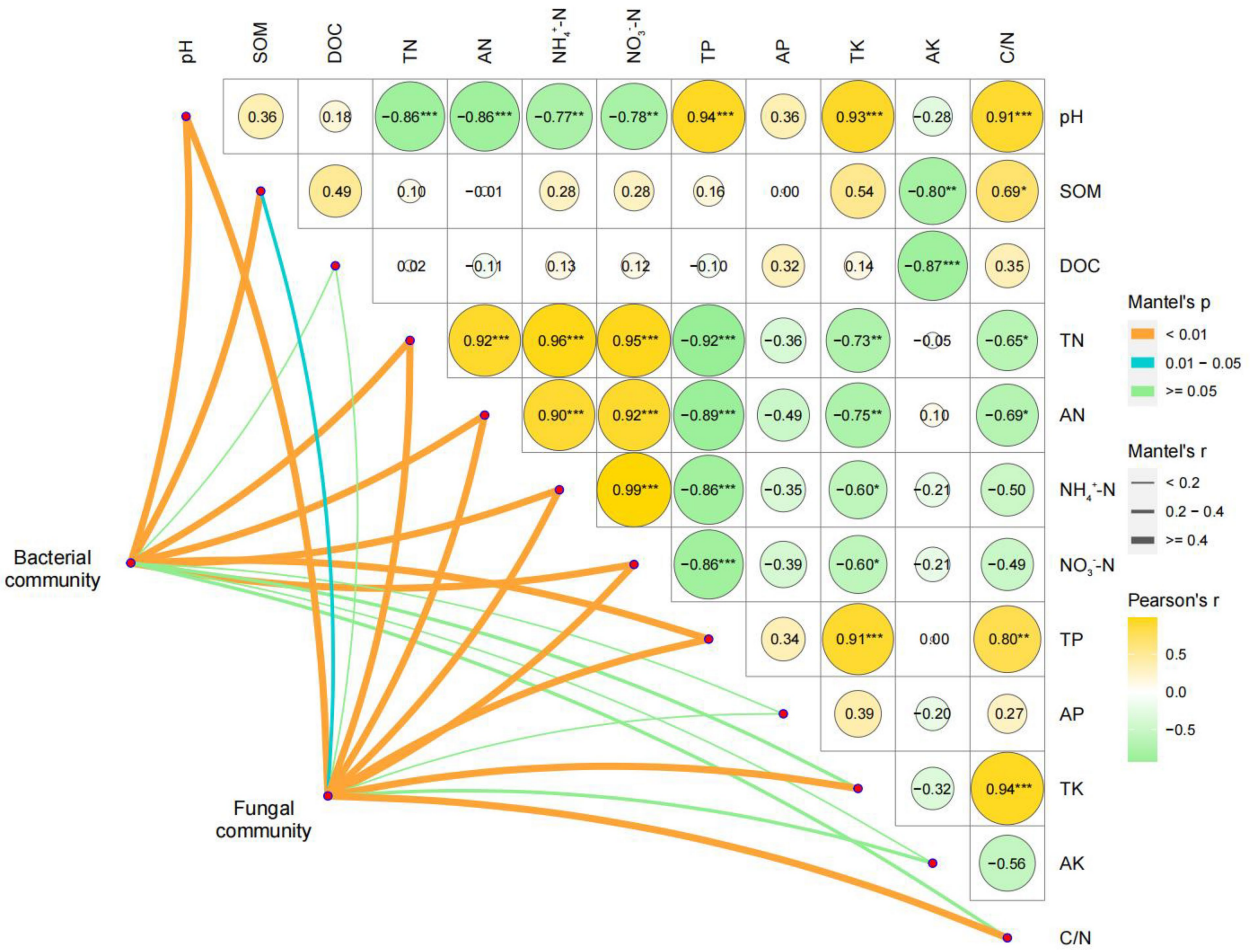
Mantel test analysis based on the relative abundance matrix of the genera with LDA value >2.0. SOM, soil organic matter; DOC, dissolved organic C; TN, total N; AN, alkali-hydrolyzed N; NH_4_^+^–N, ammonium N; NO_3_^−^–N, nitrate N; TP, total P; AP, available P; TK, total K; AK, available K; C/N, ratio of C and N.

The Mantel test was performed using the relative abundance matrix of genera in Figure 3. As shown in Figure 5 and Supplementary Table S3, pH, TN, AN, NH_4_^+^–N, NO_3_^−^–N, and TP had highly significant effects on the bacterial and fungal communities, while DOC, AP, and AK had insignificant effects. SOM had significant effects on fungal communities and highly significant effects on bacterial communities. The effects of TK and C/N on bacterial communities were not significant, but the effects on fungal communities were highly significant.

**Figure 6:**
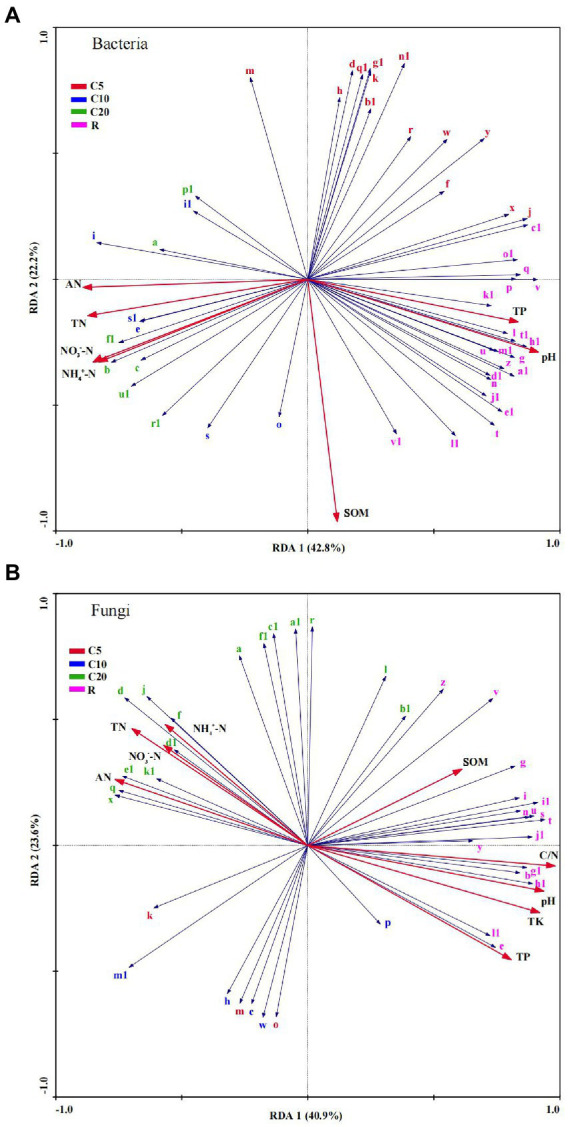
Redundancy analysis of bacterial **(A)** and fungal **(B)** communities and soil chemical properties. The generic name is the same as in Figure 3. SOM, soil organic matter; TN, total N; AN, alkali-hydrolyzed N; NH_4_^+^–N, ammonium N; NO_3_^−^–N, nitrate N; TP, total P; TK, total K; C/N, ratio of C and N.

RDA was performed based on the LEfSe and Mantel test results, combined with the VPA results. In the bacterial communities (Figure 6A), the genera of R and a few genera of C5 were positively correlated with pH (r^2^ = 0.182) and TP (r^2^ = 0.226). The genera of R and most genera of C5 were negatively correlated with TN (r^2^ = 0.375), AN (r^2^ = 0.291), NH_4_^+^–N (r^2^ = 0.363), and NO_3_^−^–N (r^2^ = 0.385), while the genera of C10 and C20 showed the opposite relationship with chemical indexes as those of R. SOM (r^2^ = 0.188) was positively correlated with most genera of C10, C20, and R, while it was negatively correlated with all genera of C5.

Similarly, in the fungal communities (Figure 6B), the genera in C5 and C10 were mostly negatively correlated with all chemical indexes. The C20 genera were positively correlated with TN (r^2^ = 0.504), AN (r^2^ = 0.557), NH_4_^+^–N (r^2^ = 0.379), and NO_3_^−^–N (r^2^ = 0.399) and were negatively correlated with pH (r^2^ = 0.638), SOM (r^2^ = 0.735), TP (r^2^ = 0.581), TK (r^2^ = 0.610), and C/N (r^2^ = 0.554), while the R genera showed the opposite pattern.

## Discussion

### Changes in microbial community diversity and composition

In this study, we used the Illumina MiSeq high-throughput sequencing method to analyze the diversity and composition of bacterial and fungal communities in tillage layer soil under soybean continuous and rotational cropping and found that the community richness indexes were significantly changed with increasing years of soybean continuous cropping, while the changes in the community diversity indexes were not significant. It was also located in the black soil region of northeastern China, Li et al. (2010) suggested that the differences in the Shannon index of bacterial and fungal communities were not significant under soybean continuous and rotational cropping, respectively, while Zhu et al. (2014) concluded that continuous cropping led to a decrease in the Shannon index of bacterial communities, with bacteria being more abundant under rotational cropping. Zhu et al. (2017) showed that continuous cropping increased both the Chao1 and Shannon indexes of the bacterial community. Liu H. et al. (2019) suggested that long-term continuous cropping did not result in significant differences in the Chao1 and Shannon indexes of fungal communities, while the Shannon index of continuous cropping was significantly higher than that of rotational cropping. Liu Z. et al. (2020) suggested that continuous cropping led to an increase in the bacterial Shannon index, while the difference between long-term continuous and rotational cropping was not significant, and the difference in the fungal Shannon index was not significant. Later, Song et al. (2022) reported that the change in the Shannon index of the bacterial community under continuous cropping was not significant, but that of the fungal community was significantly lower under the former treatment than under rotational cropping. The results of this study supported to some extent the conclusions of Zhu et al. (2017) and Liu Z. et al. (2020) and showed that bacterial community diversity was more susceptible to the influence of cropping patterns.

The dominant taxa of the soil microbial community can differ depending on cropping patterns, fertilizer management, and crop development period (Sugiyama et al., 2014; Hu et al., 2017; Hsiao et al., 2019). In this study, we found that the soil bacterial and fungal community compositions (at the OTU level) in the continuous cropping treatments differed from those in R, while their dominant phyla and genera were basically the same. The dominant bacterial phyla in all treatments were Actinobacteriota, Proteobacteria, and Acidobacteriota, which was close to the results of Liu Z. et al. (2020). However, there were differences in their relative abundance for example, Zhu et al. (2014) concluded that the relative abundance of Actinobacteriota in rotational cropping was much lower than that of Proteobacteria and Acidobacteriota, and Firmicutes had high relative abundance, while Song et al. (2022) concluded that the relative abundance of Proteobacteria and Chloroflexi increased significantly and that of Actinobacteriota and Gemmatimonadetes decreased significantly under soybean long-term continuous cropping. The results of this study differed from these findings.

The dominant fungal phyla in this study were Ascomycota, Mortierellomycota, and Basidiomycota. Liu H. et al. (2019) and Liu Z. et al. (2020) reported that Zygomycota was also a dominant fungal phylum, while the relative abundance of Mortierellomycota was extremely low or absent. However, Zygomycota was not found in this study. Liu Z. et al. (2020) showed that the relative abundance of Ascomycota did not differ significantly among treatments and that of Basidiomycota was high in rotational cropping. Song et al. (2022) found that the relative abundance of Ascomycota and Mortierellomycota increased significantly, while the relative abundance of Basidiomycota decreased significantly because of soybean continuous cropping. In this study, the relative abundance of Ascomycota increased with increasing years of continuous cropping, while that of Mortierellomycota and Basidiomycota did not differ significantly among treatments. Even in the same black soil region of northeastern China, the changes in the dominant bacterial and fungal phyla and their relative abundances differed depending on the soybean cropping patterns and the number of years of continuous cropping, and consequently, the changes in the dominant genera and their relative abundance differed from the results of previous studies.

### Beneficial and harmful microbes

In this study, *Gaiella*, *Sphingomonas*, and *Bacillus* had high relative abundance except for the unclassified taxa. It has been shown that the three genera have antagonistic effects on plant pathogenic fungi (White et al., 1996; Cutting, 2011; Cui et al., 2019). The relative abundance of *Gaiella* in C5 was significantly higher than that in R, and the relative abundance of the three genera tended to decrease from C5 to C20, but there were no significant differences between C20 and R. *Rubrobacter*, *Bradyrhizobium*, *Arthrobacter*, *Nitrospira*, and *Gemmatimonas* can participate in soil N cycling (Xu et al., 1995; Daims et al., 2015; Chee-Sanford et al., 2019; Prasad et al., 2021; Zhang et al., 2022), which is beneficial to soybean growth. In this study, only the relative abundance of *Arthrobacter* was significantly lower than that in R under continuous cropping treatments, while the relative abundances of the remaining four genera in C20 were not significantly different from those in R. It is evident that soybean long-term continuous cropping did not produce significant changes in the relative abundance of beneficial bacteria in a dominant position compared to short-term continuous cropping and rotational cropping.

Neupane et al. (2021) found that *Solicoccozyma* and *Tausonia* were enriched in the high-yielding group of soybean rotational cropping. In this study, the relative abundance of *Solicoccozyma* was significantly higher in C10 and significantly higher than that in R. In contrast, the relative abundance of *Tausonia* did not differ significantly among the continuous cropping treatments and was significantly lower than that in R. The relative abundance of *Trichocladium* was significantly increased from C5 to C20 and was significantly higher than that in R. *Trichocladium* is often considered a plant pathogenic fungus and correlates with soybean cyst nematode density (Carris et al., 1989; Nan et al., 1992). *Neocosmospora* is also a plant pathogenic fungus (Kamali-Sarvestani et al., 2022), and its relative abundance was not significantly different among C5, C10 and C20, but it was significantly higher in C20 than in R. This suggested that long-term continuous cropping may cause enrichment of *Trichocladium* and *Neocosmospora* and lead to soybean disease development.

In the present study, the relative abundance of *Fusarium* showed a decreasing trend with continuous cropping years, although the difference was not significant, but it was significantly lower in C10 and C20 than in R. *Fusarium* is considered a plant pathogenic fungus that causes root rot (Bai et al., 2015; Kamali-Sarvestani et al., 2022). Previous studies on changes in the relative abundance of *Fusarium* in soybean continuous cropping soils in the black soil region of northeastern China have presented different conclusions: Wei et al. (2015) concluded that soybean continuous cropping for 21 years was able to reduce the severity of root rot and that *Fusarium* population density was significantly lower than in rotational cropping. Liu Z. et al. (2019) and Liu Z. et al. (2020) similarly concluded that the relative abundance of soil *Fusarium* was reduced in soybean continuous cropping (13 years) and soybean-corn rotational cropping (5 years), which could alleviate the obstacles imposed by soybean continuous cropping. However, Liu H. et al. (2019) and Liu H. et al. (2020) reported that the relative abundance of *Fusarium* in soil was significantly higher in soybean long-term continuous cropping (27 years). Additionally, Song et al. (2022) suggested that soybean continuous cropping (13 years) resulted in significantly higher relative abundance of *Fusarium* and increased the incidence of root rot. The results of this study indicate that long-term continuous cropping did not lead to *Fusarium* accumulation, which to some extent can lead to disease suppressive, as proposed by Chen et al. (2018).

The function of fungi was predicted based on FUNGuild (Nguyen et al., 2015) and further screened for the taxa with significant differences among treatments. The relative abundance of the non-plant pathogenic fungi *Bifiguratus* sp., *Chaetosphaeria vermicularioides*, *Thelonectria rubrococca*, and *Phallus rugulosus* in this study tended to increase with continuous cropping years. However, *Thelonectria rubrococca* has been reported to cause plant diseases in a few cases (Salgado-Salazar et al., 2016), and *Phallus rugulosus* enrichment in soil can cause soybean diseases (Srour et al., 2017). In this study, nine plant pathogenic fungi showed significant differences among treatments. For example, *Lectera* sp., *Plectosphaerella* sp., *Volutella* sp. accumulated with continuous cropping years, while *Gibberella intricans*, *Leptosphaeria sclerotioides*, and *Paraphoma radicina* had significantly higher sequence numbers in R, which may cause soybean diseases and requires further study.

### Factors influencing microbial communities

The physicochemical properties of agricultural soils often vary depending on field management practices and cause changes to the structure and diversity of microbial communities. For example, Ma et al. (1997) reported a decrease in soil pH due to soybean continuous cropping, while Strom et al. (2020) and Liu Z. et al. (2020) suggested an increase in pH. In this study, pH was found to decrease significantly with continuous cropping years. Wang et al. (2019) and Liu Z. et al. (2020) concluded that soil pH is more important than nutrients in shaping bacterial communities. This study confirmed that soil pH had a highly significant effect on both bacterial and fungal communities.

The results of this study were consistent with those of Song et al. (2018) who suggested that continuous cropping increased the SOM content; meanwhile, the SOM content of continuous cropping in this study was significantly higher than that of rotational cropping, while Chen et al. (2018) suggested that continuous cropping led to a lower SOM content. In addition, the SOM content in this study had significant effects on both bacterial and fungal communities, and the effects on bacterial communities were higher.

Soybean continuous cropping is beneficial to the accumulation of AN content in soil (Song et al., 2018). In this study, the content of N tended to increase with continuous cropping years, which should be related to symbiotic N fixation by soybean rhizobia, and the effect on bacterial and fungal communities was highly significant. Liu Z. et al. (2020) also showed that TN content and AN content were significantly increased in soybean long-term continuous cropping systems, but they were lower than those in rotational cropping systems. Song et al. (2018) suggested that soybean continuous cropping did not fix AP, while rotational cropping was beneficial to AP content accumulation. Liu Z. et al. (2020) suggested that soybean continuous cropping led to a significant increase in soil TP and AK contents. This study showed that TP, TK, and AK contents decreased with continuous cropping years, which may be related to the depletion of soil P and K due to soybean long-term continuous cropping. In addition, the effects of P and K contents on bacterial and fungal communities in this study were different from the results of Liu Z. et al. (2020). In this study, C/N did not differ significantly among the continuous cropping treatments and was significantly lower in continuous cropping than in rotational cropping. C/N had a highly significant effect on fungi, which is consistent with the results of Liu Z. et al. (2020).

### Factors affecting soybean yield

The present study results showed that soybean yield was reduced in short-term continuous cropping compared to R, while the yield in long-term continuous cropping was close to that of R. Soybean continuous cropping increases the abundance of harmful and beneficial soil microorganisms, which in turn affects crop yield (Liu and Herbert, 2002). In contrast to conventional rotational cropping, Xu et al. (1999) suggested that soybean yield decreased by 18.6 and 35.4% in 1 and 2 years of continuous cropping, respectively. Later, Liu and Yu (2000) suggested that the average yield of soybean decreased by 9.9, 13.8, and 19% at different locations for 1, 2, and 3 years of continuous cropping, respectively. This indicates that soybean yield decreases under short-term continuous cropping.

**Figure 7:**
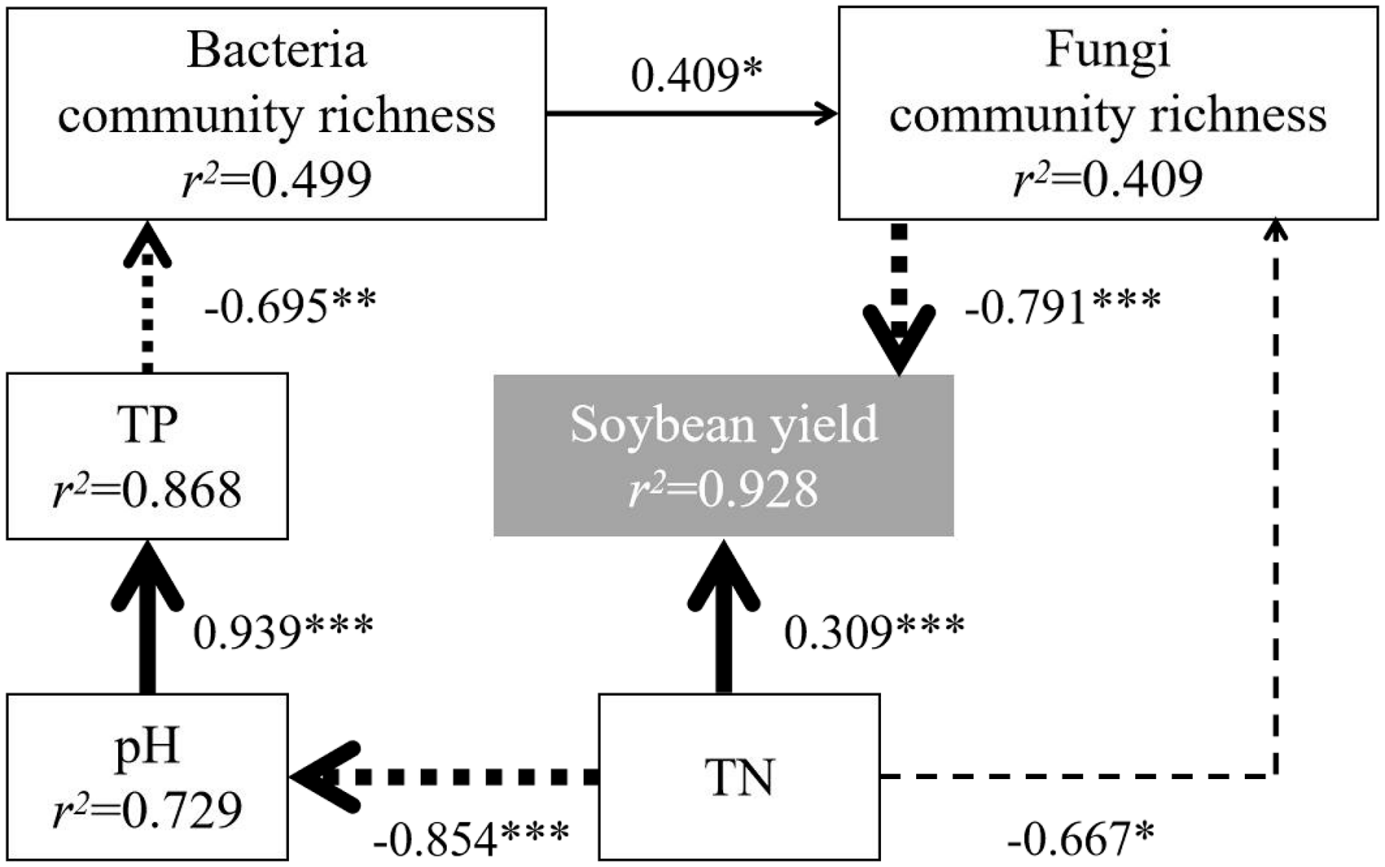
Effect analysis of bacterial and fungal communities and chemical indexes on yield. χ^2^/df = 0.662, *p* > 0.05, goodness-of-fit index >0.90, normed fit index >0.90, comparative fit index >0.90, root square mean errors of approximation <0.05, Akaike information criterion = 39.325, bootstrap = 1,000. The solid line represents positive correlation, the dotted line represents negative correlation, and the wider the line, the stronger the significance. *p < 0.05, **p < 0.01, and ***p < 0.001. TN, total N; TP, total P.

A structural equation model was fitted to analyze the effects on soybean yield using the richness index and chemical indexes with significant correlations with the bacterial and fungal communities. After model correction, the paths with significant effects were shown in Figure 7. The results showed that soybean yield was directly influenced by TN and fungal community richness and indirectly influenced by pH, TP and bacterial community richness, which was similar to the results of Strom et al. (2020). Moreover, both bacterial and fungal community richness showed negative effects on soybean yield, with total effects of-0.323 and-0.791, respectively, i.e., high bacterial and fungal community richness decreased yield, and the negative effect from changes in fungal community richness was greater. Fungal community richness was significantly higher in C5, which correlated with its low yield.

## Conclusion

Soybean continuous cropping led to significant changes in the richness indexes of the bacteria and fungi in the tillage layer soil, while the changes in the community diversity indexes were not significant. The composition of the soil bacterial and fungal communities under continuous cropping differed from that under soybean-corn rotational cropping at the OTU level, but their dominant phyla and genera were basically the same. Long-term continuous cropping did not change the relative abundance of dominant beneficial bacteria, but increased the relative abundance of harmful fungi, except *Fusarium*. Different cropping patterns resulted in changes in soil chemical indexes, where pH, SOM content, and AN content, etc., had significant effects on bacterial and fungal communities. Soybean yield was significantly correlated with TN, TP, and pH, and bacterial and fungal community richness had a negative effect on yield, with the fungal effect being greater. Soybean continuous and rotational cropping changes the soil chemical properties and drives changes in the richness of soil microbial communities, which affects soybean yield.

## Data availability statement

The datasets presented in this study can be found in online repositories. The names of the repository/repositories and accession number(s) can be found in the article/Supplementary material.

## Author contributions

YL and CS designed and performed the experiment and prepared this manuscript. DW designed this experiment. SC, YH, and WW helped to collect the soil samples and determine the chemical properties. XG and LJ helped to determine the yield and revised this manuscript. YW and LS revised this manuscript. All coauthors contributed to manuscript editing. All coauthors contributed to manuscript editing. All authors have read and agreed to the published version of the manuscript.

## Funding

This work was supported by China Ministry of Science and Technology Basic Resources Investigation Project (2021FY100406), China Agriculture Research System of MOF and MARA (CARS-04-PS17), UNDP Project (cpr/21/401), and Heilongjiang Province Key Laboratory of Cold Region Wetland Ecology and Environment Research Laboratory Open Project (201906).

## Conflict of interest

The authors declare that the research was conducted in the absence of any commercial or financial relationships that could be construed as a potential conflict of interest.

## Publisher’s note

All claims expressed in this article are solely those of the authors and do not necessarily represent those of their affiliated organizations, or those of the publisher, the editors and the reviewers. Any product that may be evaluated in this article, or claim that may be made by its manufacturer, is not guaranteed or endorsed by the publisher.

## Footnotes

^1^ http://drive5.com/uparse/
