# Supplementary material for: Soybean continuous cropping affects yield by changing soil chemical properties and microbial community richness
Source: Front Microbiol. 2022 Dec 30;13:1083736. doi: 10.3389/fmicb.2022.1083736 (PMC9838197; doi:10.3389/fmicb.2022.1083736)
Supplement: Supplementary file 1 [file Data_Sheet_1.PDF]

Supplementary Table S1 Variance analysis of the relative abundance of dominant phyla and genera (top 10 except unclassified taxa) in soybean continuous and rotational cropping soils. Data represent the means±standard deviations. Duncan's multiple comparison test was used to test the significance of differences. The different lowercase letters represent significant differences (P <0.05). C5, C10, and C20 represent soybean continuous cropping treatments for 5 years, 10 years, and 20 years, respectively, and R represents soybean–maize rotational cropping treatment.

		**Dominant taxa**	**Continue cropping**			**R (%)**
			**C5 (%)**	**C10 (%)**	**C20 (%)**	
**Bacteria**	**Phyla**	Actinobacteriota	38.94±4.17 a	36.10±2.88 ab	32.59±2.28 b	35.16±0.85 ab
		Proteobacteria	19.10±2.55 ab	14.94±1.03 b	16.68±1.96 ab	19.98±2.74 a
		Acidobacteriota	12.30±4.97 a	18.41±3.17 a	19.56±6.37 a	16.00±3.02 a
		Chloroflexi	11.48±2.75 a	12.10±1.11 a	11.93±1.70 a	10.47±0.18 a
		Gemmatimonadota	6.52±0.60 a	5.40±0.30 b	5.70±0.49 ab	5.81±0.61 ab
		Firmicutes	4.20±1.82 a	4.37±0.83 a	3.58±0.33 a	3.89±0.17 a
	**Genera**	*Gaiella*	4.65±0.65 a	3.97±0.57 ab	3.88±0.58 ab	3.40±0.48 b
		Sphingomonas	3.56±0.44 a	2.45±0.33 b	2.71±0.63 ab	3.61±0.69 a
		Bacillus	2.63±1.18 a	2.93±0.53 a	2.26±0.30 a	2.33±0.07 a
		Rubrobacter	1.36±0.37 b	1.96±0.23 a	1.62±0.20 ab	1.70±0.02 ab
		Microlunatus	1.65±0.33 a	1.70±0.26 a	1.47±0.25 a	1.88±0.15 a
		Bradyrhizobium	1.63±0.42 a	1.21±0.08 a	1.50±0.23 a	1.60±0.16 a
		Arthrobacter	1.06±0.24 b	1.15±0.25 b	1.07±0.16 b	2.61±0.69 a
		Gemmatimonas	1.26±0.31 a	0.71±0.10 b	0.83±0.20 ab	1.20±0.24 a
		Nitrospira	0.98±0.16 a	0.99±0.08 a	1.07±0.10 a	1.07±0.09 a
		Blastococcus	1.24±0.38 a	0.89±0.22 ab	0.58±0.11 b	1.07±0.20 a
**Fungi**	**Phyla**	Ascomycota	53.80±4.75 b	51.82±3.92 b	61.75±0.79 a	60.68±1.93 a
		Mortierellomycota	28.13±7.13 a	29.07±7.53 a	18.55±3.92 a	19.31±1.79 a
		Basidiomycota	12.30±5.38 a	15.47±4.68 a	15.75±2.60 a	16.48±3.45 a
	**Genera**	*Mortierella*	28.12±7.13 a	28.45±7.82 a	18.48±3.92a	19.09±1.57 a
		Solicoccozyma	6.23±0.65 b	10.41±3.18 a	12.13±1.40 a	5.31±0.75 b
		Chaetomium	7.88±1.39 a	6.66±2.16 a	6.98±1.61 a	4.79±0.64 a
		Cephalotrichum	7.71±2.00 a	7.58±3.83 a	5.68±1.72 a	5.20±4.13 a
		Fusarium	3.78±0.68 ab	3.33±1.66 b	3.33±0.95 b	5.67±0.79 a
		Trichocladium	2.58±0.66 b	2.22±0.45 bc	4.75±0.75 a	1.34±0.21 c
		Tausonia	2.78±1.82 b	1.45±1.66 b	0.74±0.30 b	9.19±2.70 a
		Paracylindrocarpon	2.61±0.63 a	2.63±0.51 a	2.43±0.99 a	1.67±0.53 a
		Pseudocoleophoma	1.15±1.02 a	0.90±0.24 a	2.72±1.67 a	1.38±0.22 a
		Neocosmospora	1.89±0.73 ab	1.65±0.51 ab	2.63±0.87 a	0.85±0.30 b

Supplementary Table S2 Taxon, taxon level, tropical mode and guild of plant-related OTUs with significant differences among different soybean cropping treatments.

OTU ID	Taxonomy	Trophic mode	Guild
OTU611	*Bifiguratus* sp.	Saprotroph-Symbiotroph	Endophyte-Soil Saprotroph
OTU210	*Chaetosphaeria vermicularioides*		Endophyte–Litter Saprotroph–Wood Saprotroph
OTU770	*Chaetosphaeria* sp.		
OTU406	Helotiaceae sp.		Ectomycorrhizal–Fungal Parasite–Plant Pathogen–Wood Saprotroph
OTU464	*Lectera* sp.	Pathotroph	Plant Pathogen
OTU665	*Plectosphaerella* sp.		
OTU279	*Volutella* sp.		
OTU695	*Gibberella intricans*		
OTU1267	*Leptosphaeria sclerotioides*		
OTU276	*Paraphoma radicina*	Pathotroph-Saprotroph	Fungal Parasite-Plant Pathogen-Plant Saprotroph
OTU1174	*Setophoma* sp.		
OTU436	*Setophoma* sp.		
OTU615	*Thelonectria rubrococca*	Saprotroph	Leaf Saprotroph
OTU51	*Phallus rugulosus*		Litter Saprotroph–Soil Saprotroph–Wood Saprotroph

Supplementary Table S3 Mantel test analysis results based on the relative abundance matrix of the genera with LDA > 2.0. SOM: soil organic matter, DOC: dissolved organic C, TN: total N, AN: alkali-hydrolyzed N, NH_4^+–N: ammonium N, NO_3^-–N: nitrate N, TP: total P, AP: available P, TK: total K, AK: available K, C/N: ratio of C and N.

Indexes	Bacteria		Fungi	
	r	*P*	r	*P*
pH	0.473	0.005	0.837	0.001
SOM	0.501	0.005	0.335	0.016
DOC	-0.109	0.782	0.164	0.087
TN	0.562	0.003	0.578	0.003
AN	0.503	0.002	0.711	0.001
NH_4^+–N	0.565	0.003	0.501	0.006
NO_3^-–N	0.596	0.003	0.530	0.005
TP	0.419	0.010	0.734	0.001
AP	0.037	0.372	0.058	0.327
TK	0.225	0.099	0.742	0.001
AK	0.093	0.212	0.231	0.070
C/N	0.277	0.057	0.711	0.001

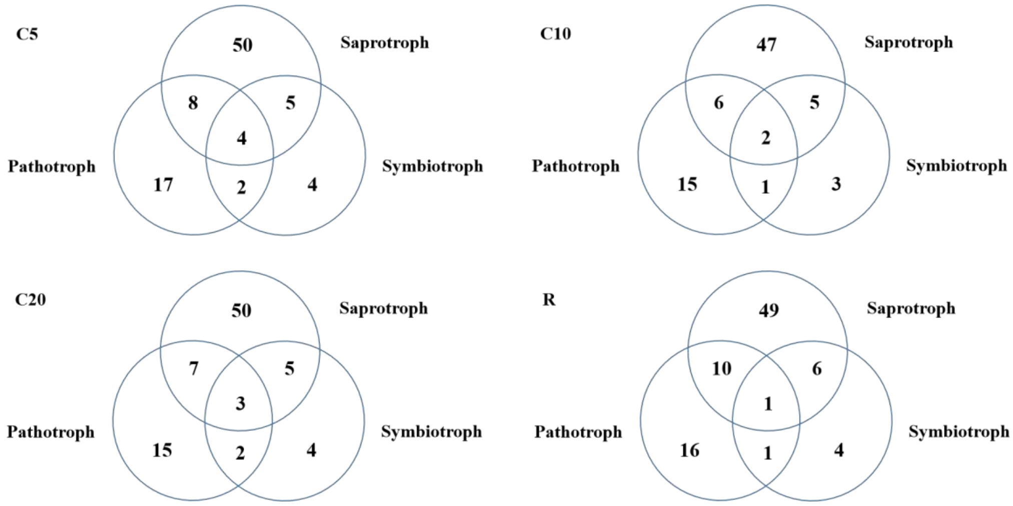

Supplementary Figure S1 Venn diagram of the number of OTUs included in the tropical mode of soil fungi in each cropping treatment. C5, C10, and C20 represent soybean continuous cropping treatments for 5 years, 10 years, and 20 years, respectively, and R represents soybean-corn rotational cropping treatment.
